# The Potential Causes of Cystic Fibrosis-Related Diabetes

**DOI:** 10.3389/fendo.2021.702823

**Published:** 2021-07-30

**Authors:** Lise Coderre, Lyna Debieche, Joëlle Plourde, Rémi Rabasa-Lhoret, Sylvie Lesage

**Affiliations:** ^1^Immunology-Oncology Section, Maisonneuve-Rosemont Hospital Research Center, Montréal, QC, Canada; ^2^Département de médecine, Université de Montréal, Montréal, QC, Canada; ^3^Division of Cardiovascular and Metabolic Diseases, Institut de recherche clinique de Montréal, Montréal, QC, Canada; ^4^Département de nutrition, Université de Montréal, Montréal, QC, Canada; ^5^Cystic Fibrosis Clinic, Centre Hospitalier de l’Université de Montréal (CHUM), Montréal, QC, Canada; ^6^Département de microbiologie, infectiologie et immunologie, Université de Montréal, Montréal, QC, Canada

**Keywords:** diabetes, cystic fibrosis, risk factors, immune function, CFTR

## Abstract

Cystic fibrosis (CF) is a genetic disease caused by mutations in the cystic fibrosis transmembrane conductance regulator gene (*CFTR*). Cystic fibrosis-related diabetes (CFRD) is the most common comorbidity, affecting more than 50% of adult CF patients. Despite this high prevalence, the etiology of CFRD remains incompletely understood. Studies in young CF children show pancreatic islet disorganization, abnormal glucose tolerance, and delayed first-phase insulin secretion suggesting that islet dysfunction is an early feature of CF. Since insulin-producing pancreatic β-cells express very low levels of CFTR, CFRD likely results from β-cell extrinsic factors. In the vicinity of β-cells, CFTR is expressed in both the exocrine pancreas and the immune system. In the exocrine pancreas, CFTR mutations lead to the obstruction of the pancreatic ductal canal, inflammation, and immune cell infiltration, ultimately causing the destruction of the exocrine pancreas and remodeling of islets. Both inflammation and ductal cells have a direct effect on insulin secretion and could participate in CFRD development. CFTR mutations are also associated with inflammatory responses and excessive cytokine production by various immune cells, which infiltrate the pancreas and exert a negative impact on insulin secretion, causing dysregulation of glucose homeostasis in CF adults. In addition, the function of macrophages in shaping pancreatic islet development may be impaired by CFTR mutations, further contributing to the pancreatic islet structural defects as well as impaired first-phase insulin secretion observed in very young children. This review discusses the different factors that may contribute to CFRD.

## Introduction

Cystic fibrosis (CF; OMIM 219700) is the most frequent autosomal recessive disease in individuals of European ancestry with approximately 1 in 3000 births (1). This disease, for which there is no cure, is caused by mutations in the cystic fibrosis transmembrane conductance regulator (*CFTR*) gene, a cAMP-regulated anion channel responsible for chloride and bicarbonate transport (1–3). To date, more than 2000 *CFTR* variants have been identified with more than 300 variants known to directly cause CF (https://cftr2.org/mutations_history). CFTR mutations are grouped into six different classes; mutations in class I-III are considered severe while those in classes IV-VI are mild, in line with the severity of the pathology resulting from these mutations (1, 3, 4).

Mutations in CFTR cause dysregulation of ion and water transport across cellular membranes, which leads to the accumulation of dehydrated mucus and thickened secretions in organs such as the lungs and the exocrine pancreas (3, 4). Consequently, CF patients show a deterioration in pulmonary function associated with recurrent pulmonary infections, a major cause of morbidity and mortality. In the pancreas, a similar phenomenon leads to pancreatic exocrine insufficiency in the vast majority of patients, resulting in nutritional deficiency characterized by poor growth or weight gain in spite normal or increased food intake. To mitigate this nutritional deficiency, CF patients must frequently take pancreatic enzymes and vitamins. In addition to the lungs and pancreas, CFTR mutations can affect other tissues leading, for example, to male infertility.

With the advent of new treatments, the life-expectancy of CF patients has increased considerably from 10 years of age to now more than 50 years in some countries (5, 6). The prolonged life expectancy has led to the emergence of additional complications such as CF-related diabetes (CFRD). CFRD is the most common comorbidity of CF and its development has been associated with a decrease in lung function and survival (7, 8). This review will discuss the etiology of CFRD and highlight the emerging potential role of inflammation in the progression to CFRD.

## Comparison of CFRD to Other Forms of Diabetes

Diabetes is a general term encompassing metabolic diseases characterized by dysregulation of blood glucose levels. The pancreas is the primary organ responsible for regulating glucose homeostasis. It is composed of exocrine and endocrine tissues. While the exocrine pancreas produces proteolytic enzymes to facilitate digestion, the endocrine tissue, named islets of Langerhans, is dispersed as cell clusters throughout the exocrine tissue (9). Each islet contains a cluster of α, β, δ, and PP cells, producing various hormones involved in glucose homeostasis (9, 10).

Diabetes is characterized by hyperglycemia. Chronic hyperglycemia contributes directly to an increased risk of vascular diseases, renal failure, blindness, amputation, and neuropathy as well as decreased life expectancy while severe hyperglycemia can lead to coma and death (11). While the most common forms of diabetes are type 1 (T1D) and type 2 diabetes (T2D), diabetes of the exocrine pancreas (DEP) is also getting recognition as an important cause of diabetes [reviewed in (12, 13)]. In T1D, representing less than 10% of overall cases, the immune system attacks and destroys insulin-producing pancreatic β-cells (14); patients living with T1D are dependent on exogenous insulin injections to regulate glucose levels (14). T2D, representing close to 90% of overall cases, is characterized by insulin resistance, whereby tissues such as the liver, muscle, and brain do not respond efficiently to insulin resulting in impaired glucose homeostasis. In addition, T2D patients exhibit quantitative and qualitative defects in insulin production. The combined effects of insulin resistance and abnormal insulin secretion together with other important mechanisms such as dysregulated secretion and/or action of incretin hormones and kidney glucose reabsorption lead to increased blood glucose levels (15, 16). Early treatment of T2D targets three different pathways, alone or in combination by: 1) improving insulin sensitivity *via* either changes in lifestyle or pharmacologic approaches; 2) increasing insulin secretion; and 3) reducing kidney glucose reabsorption (17). Over time, the ability of pancreatic β-cells to produce insulin is reduced in most T2D patients and exogenous insulin may be required to maintain appropriate glucose levels (18). A major complication of insulin therapy is hypoglycemia, which is associated with physical (e.g. falls) and/or psychological complications (e.g. fear) and, in its most severe form, can lead to coma or death (19). DEP, also named type 3c diabetes or pancreatogenic diabetes, refers to patients that develop diabetes secondary to exocrine pancreatic diseases such as acute and chronic pancreatitis, pancreatic cancer, alcohol-induced pancreatitis and CFRD. DEP is often underdiagnosed and may represent between 1-9% of diabetes cases (20, 21). Alcohol consumption is a leading cause of DEP (22), and alcohol-induced pancreatitis is associated with the accumulation of mucus in small ductal cells and destruction of the exocrine pancreas (22, 23). Interestingly, alcohol consumption is also associated with a reduction of the expression and membrane localization of CFTR in human pancreas (24, 25), and *CFTR* variants are important risk factors for DEP (26, 27). These observations highlight the important role of CFTR in the maintenance of pancreatic functions beyond CF. Still, because of the heterogeneous nature of the underlying mechanism driving the lesions in the exocrine pancreas for DEP, these diseases tend to exhibit very different pathophysiological features (18). One common feature of various forms of DEP is the reduction of insulin secretion. Consequently, DEP patients resort to the administration of exogenous insulin to regulate their blood glucose levels.

CFRD is a heterogeneous disease that shares some characteristics of T1D, T2D and alcohol-induced diabetes, such as insulin insufficiency. Similar to other forms of DEP, CFRD is associated with pancreatic inflammation, fibrosis and fatty infiltration leading to a reduction of the number of islets and impaired insulin secretion. The development of CFRD in CF patients is not associated with β-cell autoimmunity or HLA haplotypes that are linked to T1D susceptibility (28, 29). This suggests that for a majority of CF patients, the development of diabetes is not due to an autoimmune destruction of the β-cells. Nevertheless, a small subset of CF patients present evidence of an autoimmune response directed towards insulin-producing pancreatic β-cells. Specifically, autoantibodies to β-cell antigens, such as glutamic acid decarboxylase, insulin, or protein tyrosine phosphatase, which can be present in T1D patients (30, 31), have been detected in 0.8%-8.5% of CF patients (28, 29, 32). In this subset of CF patients, diabetes onset occurs earlier and is associated with a higher risk for acute complications, such as severe hypoglycemia and ketoacidosis (32). Thus for these autoantibody-positive CF patients, the autoimmune response to β-cell antigens is associated with the progression and severity of CFRD. Whether HLA haplotypes linked to T1D susceptibility or whether autoreactive T cells are more prevalent in autoantibody-positive CF patients relative to autoantibody-negative patients remains to be determined. From a clinical standpoint, CFRD and T1D share common features: onset is mostly in young patients, the diagnosis is typically not associated with obesity and insulin therapy is very frequently the therapeutic option. Of interest, a *CFTR* variant has recently been linked to T1D susceptibility suggesting a further association between CFRD and T1D pathologies (33).

CFRD also shares some features of T2D, such as insulin resistance and pancreatic amyloid deposition (34–37). Though some older studies failed to identify insulin resistance as a component of dysglycemia in CF, most recent studies show that CFRD patients exhibit both peripheral and hepatic insulin resistance similar to T2D (37–41). The hepatic insulin resistance coupled with a higher rate of gluconeogenesis result in a net increase in hepatic glucose production, which contributes to the hyperglycemia in CF patients (38, 39). Prospective observational cohort studies show that in a context of significantly reduced insulin secretion, variations of insulin sensitivity are associated with variations of glucose tolerance in adult patients with CF (41). Pancreatic amyloid deposition is also characteristic of CFRD patients. It is observed in 69% of CFRD patients, whereas it is absent in CF patients without diabetes (35). Interestingly, amyloid deposits are also observed in diabetics suffering from chronic pancreatitis and pancreatic cancer (42). As for T2D, this amyloid deposition in CFRD is progressive; it is generally not observed in children with CF (36, 43, 44). Because these deposits are detected late in the disease process, amyloidosis is probably a consequence rather than a cause of diabetes. Moreover, a recent study has found elevated levels of inflammatory markers in T2D and CFRD patients compared to control subjects, and these markers were associated with diabetic complications in both groups of patients (45). Of note, systemic inflammation is usually associated with insulin resistance. From a clinical standpoint, the onset of diabetes in both CFRD and T2D is preceded by a long phase of glucose intolerance usually characterized by postprandial glucose excursions (46).

A most interesting commonality between T2D and CFRD is the view that lung function may contribute to glucose dysregulation. In a T2D cross sectional study, lower forced expiratory volume in one second (FEV1), a measure of lung function, was associated with higher levels of plasma glucose in both control and T2D subjects (47). Similarly, in CF children, there is an inverse association between lower arterial oxygen saturation at night and glucose excursion during the OGTT (48). Prospective studies have also reported that reduced lung function increases the risk of T2D (49, 50). It would be interesting to examine whether reduced lung function in CF accelerates the progression to CFRD.

Altogether, results from these studies suggest that CFRD is a heterogeneous disease and that progression to CFRD is regulated by a multitude of factors. A key specific point of abnormal glucose homeostasis in patients living with CF is an association with an increased risk of accelerated weight loss, lung function reduction, and accordingly, a marked increased risk of early death (51, 52). Historically, it was reported that such risks start in the pre-diabetic glucose intolerance phase (53). Recently published data, however, indicate that improvement of overall CF management could modify some of the previously reported associations. For example, CFRD onset is no longer preceded by a reduction in lung function and/or accelerated weight loss in the majority of patients (53).

## Risk Factors Associated With the Development of CFRD

Age, genotype and the presence of exocrine pancreatic deficiency are defined risk factors for CFRD. Notably, similar to insulin resistance (7, 37, 53, 54), the prevalence of CFRD increases with age; around 2% of children, 19% of adolescents, and up to 50% of adult CF patients are diabetic (7). In addition, patients carrying severe mutations such as class I and II mutations have a higher risk of diabetes and pancreatic insufficiency compared to those with milder CFTR mutations (54). Pancreatic insufficiency is closely associated with dysglycemia as well as low body mass index (BMI) and FEV1 (53). However, not all patients bearing identical CFTR mutations will develop CFRD and variation in diabetes onset as well as disease severity among CF patients with the same mutation suggest that additional factors contribute to CFRD. Genome wide association studies (GWAS) indicate that while there is only weak genetic association between T1D and CFRD (55), CFRD shares some genetic etiology with T2D, including loci associated with β-cell function (55, 56). In fact, a family history of T2D increases by 3-fold the risk of developing CFRD (56). GWAS have also highlighted the contribution of gene modifiers to CFRD. For example, variants of *SLC26A9*, a chloride channel, are associated with CFRD onset (55, 56) and patients that express higher levels of this transporter develop diabetes later (57). While *SLC26A9* variants are specific for CFRD, other variants such as *TCF7L2* are associated with both CFRD and T2D (55).

In addition to age and genotype, gender also contributes to CFRD susceptibility, but its role is more complex. Indeed, CFRD is more prevalent in women with CF in spite of higher insulin secretion compared to men with CF (58). One possible explanation for this counterintuitive observation is the fact that bacterial infections occur earlier and more frequently in women than men with CF, often leading to a systemic inflammatory response (59). Both acute and low grade systemic infections as well as corticosteroid use are known to contribute to insulin resistance and diabetes (60–63). Interestingly, Harding et al. showed that TNF-α levels, a marker of systemic inflammation, correlated with worse clinical status in CF subjects with impaired glucose tolerance (64). Altogether, this suggests that heightened systemic inflammation present in women relative to men with CF exacerbates insulin resistance and contributes to CFRD development.

CFTR mutations also affect the hepatobiliary system and liver diseases are risk factors for CFRD (65). Of note, liver biopsies from CF patients revealed fatty degeneration and liver fibrosis, which can progress to liver cirrhosis (66). Studies have shown that there is a strong association between liver steatosis and insulin resistance [reviewed in (67)]. In addition, crude markers of hepatic damage are associated with higher probability of dysglycemia and surrogate markers of insulin resistance and this effect was predominantly observed in men with CF (68). As mentioned above, CF patients present with hepatic insulin resistance and increased gluconeogenesis contributing to hyperglycemia (38, 39). The mechanism leading to CF liver diseases is not completely understood. Still, because CFTR is expressed in bile ducts and not in hepatocytes, the prevalent hypothesis is that, similar to exocrine pancreas, inspissated bile will obstruct small ducts causing liver injury and inflammation, resulting in liver fibrosis (69).

Overall, multiple factors are associated with increased risk or accelerated progression to CFRD, such as age, gender, *CFTR* genotype, genetic modifiers, the degree of pancreatic exocrine deficiency, lung function, liver disease, inflammation, the presence of β-cell specific autoantibodies, etc. Defining how the combination of these factors drives the progression to CFRD in each individual would increase our understanding of the heterogeneity of disease severity and onset.

## Glucose Homeostasis in CF Patients

In diabetic patients, hyperglycemia can be diagnosed by quantifying fasting, post-glucose charge, or random plasma glucose levels, as well as glycosylated hemoglobin (A1c), a biomarker of mean plasma glucose over 3 months (70). Diabetes is usually preceded by a phase of glucose intolerance, which can affect fasting and/or postprandial glucose values. In CF patients, abnormal glucose tolerance (AGT) is diagnosed in more than 50% of adult CF patients.

Studies of CF children suggest that the incidence of AGT do not follow a linear progression across age group. Notably, the prevalence of AGT is higher in 2-4 year old children than in those over 5 years of age, suggesting that glucose abnormalities occur very early in life and that the severity of the dysregulation of glucose homeostasis may be age-dependent. A transient increase in the prevalence of AGT has also been observed in an animal model of CF, namely, in *CFTR*-null ferrets (71). Indeed, in young *CFTR*-null ferrets pancreatic inflammation and hyperglycemia peak in two month old kits and resolve thereafter (72, 73). These changes in glucose homeostasis in *CFTR*-null ferrets correlate with the destruction of the exocrine pancreas by proteolytic enzymes and remodeling of the endocrine pancreatic tissue, including the insulin-producing β-cells (73). Based on the observations in *CFTR*-null ferrets, it is tempting to suggest that AGT in children may result from deleterious exocrine and endocrine pancreatic tissue remodeling that increases the risk of developing CFRD later in life.

AGT increases the risk of developing diabetes in CF patients, and the detection of AGT in children aged 6-10 years old is associated with early onset CFRD (74). Inversely, cross-sectional analysis of adult CF subjects showing normal glucose tolerance [i.e. with peak blood glucose levels below 8 mmol/L during an oral glucose tolerance test (OGTT)] did not develop diabetes over the next decade (75). However, it should be noted that the number of CF patients with defects in glucose homeostasis is likely to be dramatically underestimated. Studies using continuous glucose monitoring show that 75% of young children (2-6 years old) and most adults with CF exhibit some glucose abnormality, despite showing normal variations in glucose levels when subjected to an OGTT (76–78). Still, questions persist about which parameters and glucose thresholds derived from continuous glucose monitoring should be used to diagnose CFRD and also to predict the risk of adverse clinical outcome especially rapid BMI and/or FEV1 decline.

The widespread glucose abnormalities observed in CF subjects are primarily due to altered insulin secretion, especially a reduction in first-phase insulin secretion (79–81). AGT is detectable in very young infants, in line with the fact that CF patients exhibit early endocrine pancreatic abnormalities, such as β-cell dysfunction and/or reduced β-cell mass. Indeed, while there are no significant changes in pancreatic islet size and distribution, histopathological analysis of pancreas from neonates and young children reveal a reduced number of insulin-positive β-cells per islets. Among insulin-positive β-cells, there is also a decrease in β-cell proliferation and neogenesis (36). Pancreatic β-cells are thus considerably reduced in young CF patients and are not being replaced. These changes correlate with the reported abnormalities in insulin secretion in both pancreatic sufficient and insufficient CF children (82, 83). Still, the decrease in insulin production in CF patients is not reliably predictive of CFRD onset. Interestingly, the decrease in β-cells is concomitant with an increase in the proportion of glucagon- and somatostatin- positive α- and δ-cells, respectively (36). Furthermore, secretion of glucagon, somatostatin and pancreatic polypeptide is dysregulated in CF patients and may participate in abnormal glucose regulation of CFRD patients (84–86). Altogether, these results suggest that, in CF patients, the endocrine dysfunctions are not limited to β-cells; rather the function of the whole endocrine pancreas is severely affected.

## Indirect EffectS of CFTR Mutation on β-Cell Function

Earlier studies of CF were mostly from histological sections of pancreas autopsies from both CF and control subjects. They were very detailed and generated extensive descriptions of pancreatic injury. However, because they were mostly from autopsies, they tended to represent later stages of the disease or more severe mutations. More recent immunochemistry studies provide a detailed description of islet cellular composition, as well as markers of cell proliferation and β-cell neogenesis. In addition, isolation of human pancreatic islets from CF patients has allowed for more dynamic studies such as cellular electrophysiology as well as insulin secretion furthering our comprehension of CFRD. To determine whether these observations are generalizable to all CFRD patients, these studies would benefit from gaining access to larger patient cohorts, allowing, for instance, to establish the impact of mutation class, age, gender and other variables. Regardless, these studies have revealed important aspects of CFRD pathology, and we discuss them in greater detail.

**Figure 1:**
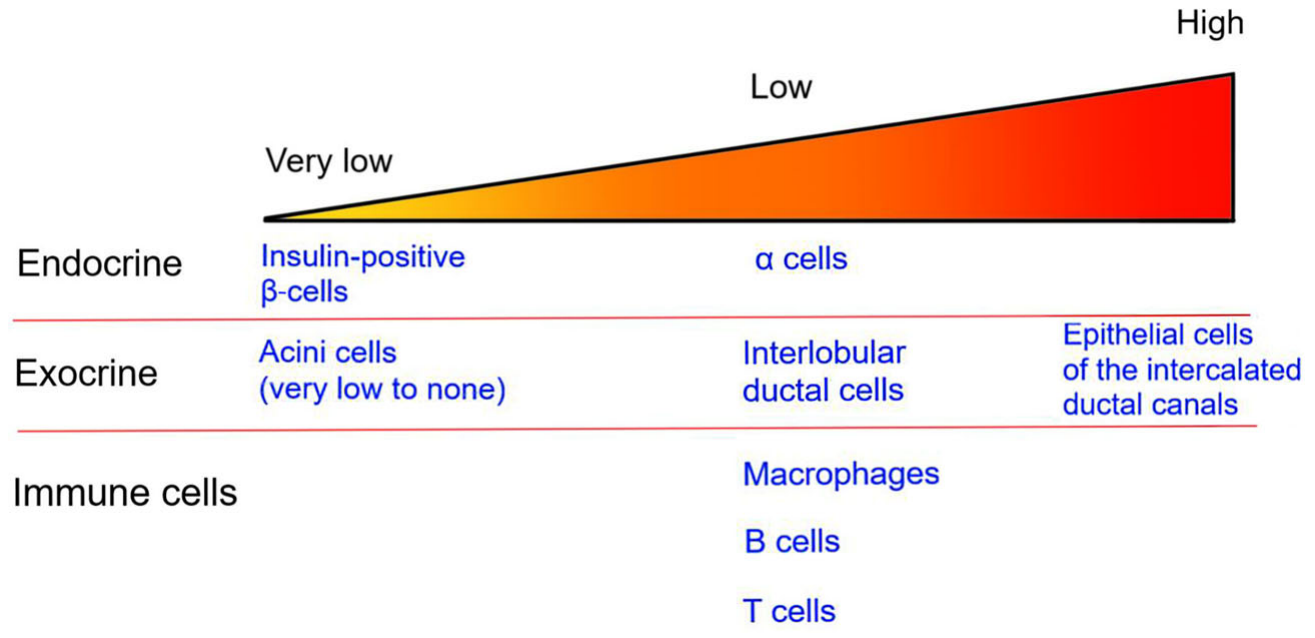
CFTR expression in the pancreas. The image represents the relative level of CFTR expression in pancreatic endocrine and exocrine tissues as well as in immune cells. Specific cell types are listed.

In the human pancreas, the CFTR protein is detected in endocrine α-cells as well as in the exocrine pancreas (**Figure 1**). However, its presence in β-cells remains controversial, with most studies reporting very low levels of CFTR expression in human β-cells (87–91). In human fetal tissues, *CFTR* mRNA expression is found in the ducts of exocrine pancreas but not directly in the pancreatic islets (89, 92). More recently, *in situ* analysis showed that *CFTR* mRNA is detectable in only 0.45% of insulin-positive β-cells, in accordance with other studies which detected low levels of *CFTR* mRNA in islets (88, 90). However, CFTR protein and activity are not detected in human β-cells (90). Together, these findings suggest that only a small fraction of human β-cells potentially express CFTR. As such, the global alteration in β-cell insulin secretion in CF patients is most likely due to the impact of CFTR mutations in non β-cells.

**Figure 2:**
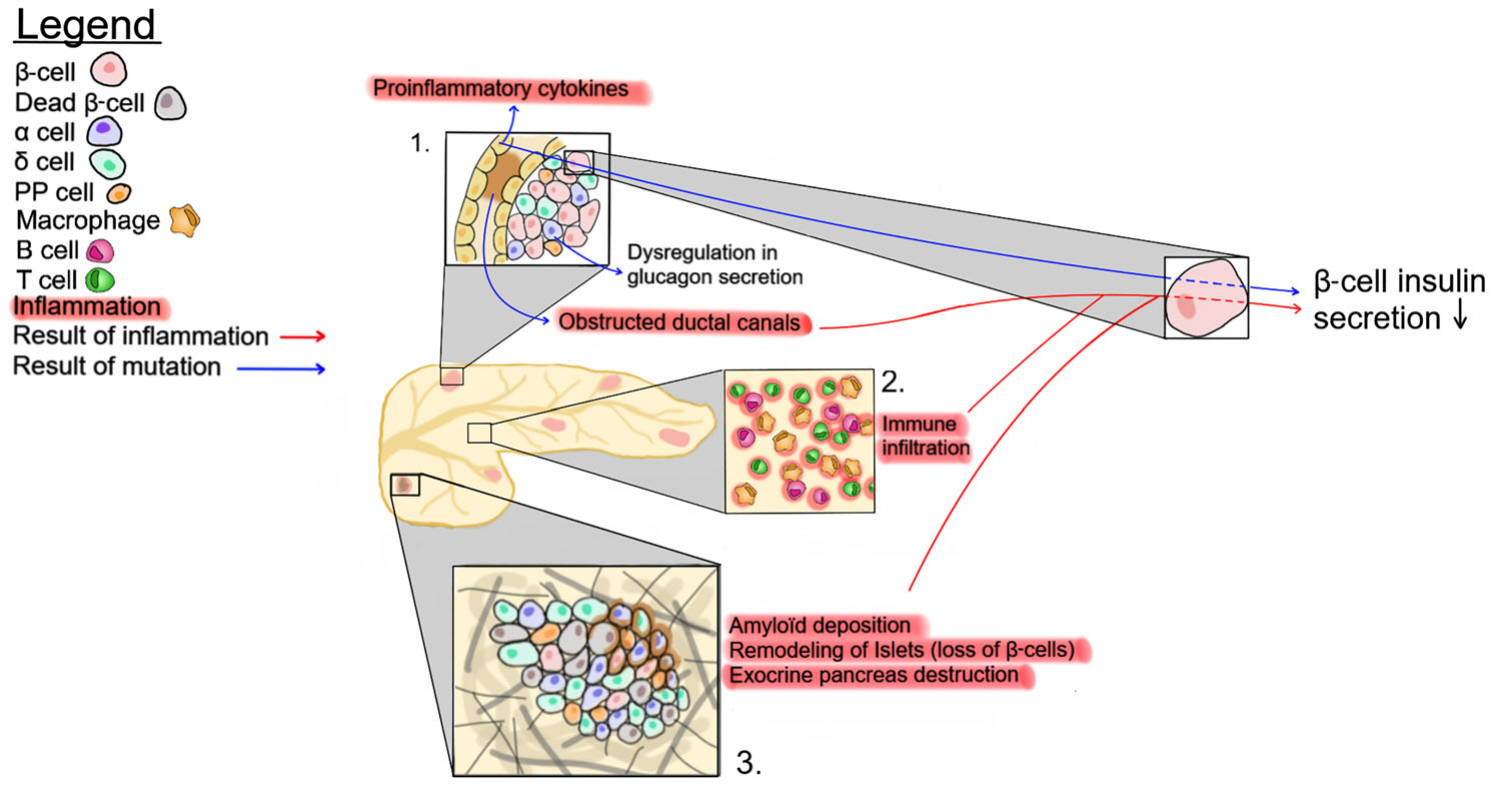
Possible mechanisms affecting insulin secretion in CF patients. 1- CFTR mutations eventually lead to ductal obstruction, which causes an increase in proinflammatory cytokines. 2- The accrued immune infiltration in the pancreas of CF patients. 3- Changes in pancreatic tissue structure, such as amyloid deposition, exocrine tissue destruction and β-cell loss. These three pathways impact insulin production by β-cells.

The islets, which represent only 1-2% of the pancreatic mass, are surrounded by the exocrine pancreas. Within the exocrine pancreas, CFTR has not been detected in the acini cells and is expressed at low levels in the interlobular duct, but at high levels in the epithelial cells of the intercalated duct canal (**Figure 1**). Notably, CFTR expression in the ductal system is detected early during embryonic development, starting at week 12 of gestation (92–94). Appropriate CFTR function is necessary for the development of the exocrine pancreas, as mutations in CFTR result in profound structural abnormalities in this tissue (95). With disease progression, the pancreatic ductal system of CF subjects shows high levels of protein concentration, such as trypsin, leading to protein precipitation and calcification. All these changes favor tubule dilation eventually provoking acini disruption and the release of pancreatic proteolytic enzymes within the pancreas (96, 97). This process, which starts in utero, continues postnatally (98). Notably, pancreatic structural abnormalities are detected at birth in CF infants (97, 99). In most CF subjects, the release of proteolytic enzymes will cause the destruction of the exocrine pancreas, followed by the activation of tissue repair mechanisms, fibrosis and the eventual replacement of the exocrine pancreas tissue by fat (**Figure 2**). This process leads to remodeling of the pancreas with islets often found clustering together (100). The destruction of the exocrine pancreas results in pancreatic insufficiency in 85% of CF patients while up to 20% of pancreatic sufficient CF patients will develop pancreatitis.

There is a close physical connection between the ductal system and pancreatic islets, as well as a strong association between markers of exocrine pancreatic damage, such as circulating immunoreactive trypsinogen, and the risk of developing CFRD (101). Recently, this association has been strengthened by the identification of genetic variants, including *PRSS1* and *SLC26A9*, coding for cationic trypsinogen and a chloride channel, respectively, as predictors of CFRD (102). This suggests that the destruction of the exocrine pancreas and the resulting inflammation and fibrosis may contribute to disorganization, dysfunction, and destruction of the endocrine tissue (**Figure 2**). Accordingly, early histological examinations of CF pancreas by Andersen and others, described the presence of fibrosis as well as of cysts in infant pancreatic tissues at autopsy (96, 99). However, a recent study by Bogdani et al. show some fibrosis but very little fatty infiltration in the pancreas of children under 4 years of age (36) while CF adults show islet sclerosis and reduced β-cell areas (36, 100). It should be noted that results from older CF patients with severe pancreatic disease seem to separate into two broad phenotypes, a more fibrotic phenotype or one with a lipoatrophic pattern i.e. fat tissue enveloping pancreatic islets (36, 100). At present, it is unclear whether *CFTR* genotype, modifier genes or other factors play a role in the generation of these different patterns but they seem to be independent of CFRD (36, 100). It is also possible that, similar to the *CFTR*-null ferret, fibrosis will ultimately be followed by fatty infiltration (73). Altogether, these observations suggest that mutations in CFTR lead to alterations in the exocrine pancreas resulting in inflammation and tissue repair, which consequently modulate the pancreatic islet structure.

The sum of these correlative studies is not sufficient to determine if defective CFTR function in pancreatic ductal cells can directly influence insulin secretion by pancreatic β-cells. To address this, Shik Mun et al. designed a two-chamber *in vitro* system, wherein they cultured pancreatic ductal epithelial cells and islets in two interconnected chambers. Interestingly, chemical inhibition of CFTR function had no direct effect on isolated islet cells. However, it specifically affected the function of ductal cells, which resulted in a 50% decrease in insulin secretion by pancreatic islet cells (103). This study demonstrates that inhibition of CFTR function in ductal cells impedes insulin secretion likely through soluble mediators.

Findings from the latter study exploiting a two-chamber *in vitro* system suggest that improving CFTR function in ductal cells may ameliorate glucose homeostasis in CF patients (103). This has been addressed somewhat clinically through the administration of novel drugs, namely lumacaftor, a corrector that binds to and improves CFTR folding and expression and ivacaftor, a potentiator, which enhances CFTR function by facilitating channel opening. Early studies with ivacaftor alone suggested that this treatment may have a beneficial effect on glucose tolerance and the development of CFRD (104, 105). However, in more recent studies, CF patients treated with both lumacaftor and ivacaftor did not improve glucose tolerance or insulin secretion (106–108). It could be that administration of the drugs occurred too late in the disease process since most of the pancreatic CF manifestations are already present at birth. Future studies should investigate the effect of lumacaftor/ivacaftor in very young children and/or over a longer time-period.

In addition to their role in bicarbonate production and pH maintenance, pancreatic ductal epithelial cells have the capacity to secrete various growth factors and cytokines/chemokines, which may be affected by CFTR mutations (**Figure 2**). Ductal cells constitutively express CD40, and *in vitro* activation of purified pancreatic ductal epithelial cells with CD40L increases the secretion of multiple inflammatory cytokines and chemokines, including MIP-1β, IL-6, IL-8, TNF-α, IL-1β, IFN-γ, and granulocyte-macrophage colony-stimulating factor (GM-CSF). In addition, similar to the morphological changes of the pancreas (fibrosis; fat infiltration), the composition of the leukocytic infiltration was found to change over time, being composed primarily of macrophages and T lymphocytes in young patients, and of T lymphocytes with very few, if any, macrophages in adult CF patients at a time when the destruction of the exocrine pancreas is almost complete and remodeling of the endocrine pancreas has occurred (36, 43). This is consistent with the role of macrophages and lymphocytes in inflammation, tissue remodeling and fibrosis which is predominant in young children pancreas (109). These observations suggest that the inflammatory response in CF patients is a dynamic process that may contribute to the pathology.

## Possible Contribution of Immune Cells in Progression to CFRD in CF Patients

Although CFTR is expressed at low levels in immune cells (**Figure 1**), CFTR mutations are associated with alterations of both innate and adaptive immune responses in CF subjects (110–112). Thus, in addition to the inflammation generated by the destruction/damage of the exocrine pancreas, dysregulation of the immune system may contribute to impaired insulin secretion and the development of CFRD (**Figure 2**).

Macrophages are one of the immune cell types that express CFTR (113–115). Indeed, mutations in CFTR have an impact on macrophage function, arguably promoting a proinflammatory phenotype (112, 113, 116, 117). In healthy humans and in mice, macrophages are detected early in the embryonic pancreas, and they contribute to pancreatic growth as well as to the establishment of β-cells in perinatal life (118, 119). Mice deficient in macrophages show abnormal islet morphogenesis, smaller islets, and a reduction in insulin mass (118, 120). Macrophages also play an important role in angiogenesis and tissue repair mechanisms, and can increase β-cell proliferation to maintain islet mass following pancreatic damage (121, 122). As mentioned above, intra-islet infiltration of monocytes/macrophages are increased in young CF patients, but are absent in both adult CF patients and control subjects (36, 43). This suggests that the presence of macrophages in the pancreatic islet tissue is a transitory phenomenon in CF. Alterations in macrophage function as a consequence of CFTR mutations in young CF patients may impact pancreatic development and thus contribute to enhanced susceptibility to CFRD. Of note, inflammatory macrophages are also found in the pancreatic islets of T1D and T2D patients, as well as in chronic pancreatitis (123–125). In chronic pancreatitis, activated macrophages participate in lymphocyte recruitment through the secretion of chemokines as well as in the progression of fibrosis in concert with pancreatic stellate cells (126, 127).

Macrophages also regulate insulin secretion, in part through soluble mediators such as IL-1β (128). Immunoreactivity for IL-1β was observed in pancreatic islets of CFRD patients as well as in young children with CF (43). In contrast to T2D, CF islets do not express the IL-1β antagonist, IL-1Rα, suggesting that the bio-activity of IL-1β is even higher in CFRD than in T2D (43). Macrophages may thus play a general role in all forms of diabetes and pancreatic inflammatory conditions, including CFRD. Additional studies are required to assess the phenotype and function of macrophages within the pancreatic tissue of CF patients, and to define whether altered macrophage function in CF patients contributes to the development of CFRD.

In addition to macrophages, there is growing evidence that T cell responses are altered in CF patients. T cells play an important role in both T1D and T2D, suggesting that they may also contribute to CFRD. Specifically, autoreactive T cells destroy β-cells directly contributing to T1D pathology, whereas in T2D, T cells contribute to systemic inflammation (129–131). T cells are also recruited in the pancreas during acute and chronic pancreatitis [reviewed in (132)]. T cell function in CF patients has been studied primarily in the lungs, often in the context of pulmonary infections. These studies revealed a reduction in regulatory T cells, as well as an increase in cytokine and chemokine production compatible with a bias towards a Th2 and/or a Th17 phenotype (133–137). T cell production of IL-17 is notably increased in CF patients (138). This is of interest to CFRD as the abundance of Th17 cells, which produce IL-17, is elevated in the blood of both T1D and T2D patients (131, 139). The presence of Th17 cells in the blood may thus contribute to diabetic pathologies. In addition to these systemic phenotypes, CF patients have lymphocytic infiltrates in pancreatic islets (36, 90). The T cells infiltrating the pancreas in CF patients secrete proinflammatory cytokines, known to inhibit insulin secretion [reviewed in (140)] Proinflammatory T cells in CF may thus contribute to CFRD by producing cytokines that increase the level of systemic inflammation, by infiltrating islets, and by destroying pancreatic β-cells, as well as by inhibiting insulin production by producing cytokines in the vicinity of β-cells.

B lymphocytes also express CFTR, and are likely to contribute to CFRD, at least in patients presenting with autoantibodies to islet antigens. Indeed, as mentioned previously, β-cell autoantibody-positive CF patients tend to develop CFRD earlier than autoantibody-negative individuals (32). Studies in *Cftr* ^-/-^ mice suggest that CFTR mutations directly affect B lymphocyte function. Uninfected *Cftr* ^-/-^ mice have higher levels of the B cell survival factor, B cell-activating factor (BAFF), a member of TNF cytokine family, as well as an increased number of lung lymphoid follicles compared to control mice (141). Increased levels of BAFF and lymphoid follicles were also observed in CF patients (141). Moreover, the pancreas of newborn *CFTR* ^-/-^ pigs present with a higher proportion of activated B lymphocytes, likely producing antibodies. These results suggest that CFTR alters B lymphocyte homeostasis, promoting the accumulation of B cells, which may contribute to CFRD. Interestingly, in addition to β-cell autoantibodies, some CF patients also exhibit autoantibodies to actin, and to double-stranded DNA (142). Overall, these data suggest that autoimmunity is a relatively frequent occurrence in CF patients. However, whether this effect is intrinsic to B lymphocytes or a consequence of recurrent lung infections remains to be demonstrated.

## Discussion

The etiology of CFRD is complex with reduced insulin secretion playing a dominant role. However, despite numerous studies, the causal factor(s) implicated in disease onset and progression remains to be identified. Severe CFTR mutations cause alterations in the exocrine pancreas, which attract immune cells to this tissue leading to inflammation, islet disorganization, loss of β-cell mass, and reduced insulin production. The onset of exocrine pancreatic destruction in utero may explain, at least partially, why glucose abnormalities and altered insulin secretion are detected in very young infants. However, despite these changes, CFRD is rare in infants and not all CF patients progress to CFRD in adulthood, suggesting that other mechanisms contribute to disease onset. Immune pathways contribute to the normal development of the pancreas, but also to the pathogenesis of T1D and T2D and could play a similar role in CFRD. In particular, macrophages are important for normal islet homeostasis and CFTR mutations in macrophages may affect their function contributing to islet dysfunction and impaired insulin secretion. CF patients also show other alterations in immune function such as enhanced cytokine secretion by T cells and a bias toward Th2 and Th17 responses in the lung, as well as accumulation of B cells and the production of autoantibodies. These changes could contribute to the development of CFRD either directly or through their crosstalk with macrophages. Thus, despite intense research, numerous questions remain on the causes of CFRD onset in CF patients that should be the focus of future studies.

## Author Contributions

LC supervised LD and wrote the final version of the manuscript. LD wrote the first draft of the manuscript with LC. JP prepared the figures and edited the manuscript. RR-L wrote parts of the manuscript and edited the manuscript. SL supervised the study and edited the manuscript. All authors contributed to the article and approved the submitted version.

## Funding

LD: holds a Diabete-Quebec studentship. RR-L: J-A DeSève diabetes research chair and CF Canada operating grant. SL: Research Scholar Emeritus awardee from the FRQS.

## Conflict of Interest

The authors declare that the research was conducted in the absence of any commercial or financial relationships that could be construed as a potential conflict of interest.

## Publisher’s Note

All claims expressed in this article are solely those of the authors and do not necessarily represent those of their affiliated organizations, or those of the publisher, the editors and the reviewers. Any product that may be evaluated in this article, or claim that may be made by its manufacturer, is not guaranteed or endorsed by the publisher.
